# 

**Published:** 1892-06

**Authors:** 


### CONTENTS.

CLINICAL LECTURE—
Uterine Myoma—Umbilical Hernia—Amputation of the Thigh. By Thomas H. Manley, M. D., New York ........ 193
ORIGINAL COMMUNICATIONS—
Typho-Malarial Fever. By J. W. Duncan, M. D., Atlanta, Ga. ........ 198
Recent Experiences with Puerperal Septicæmia. By W. D. Haggard, M. D., Nashville, Tenn. ........ 206
Post-Partum Hemorrhage. By Roger Williams, M. D., Pittsburg, Pa. ........ 211
SOCIETY REPORTS—
The Clinical Society of Maryland ........ 218
CORRESPONDENCE—
New York Letter ........ 225
EDITORIAL—
Gall-Bladder Surgery ........ 230
Why "Heart-Failure?" ........ 233
Keeley in the Pulpit ........ 236
The Salts of Strontium ........ 237
SELECTIONS ........ 238
MEDICAL ITEMS ........ 251
BOOK REVIEWS ........ 254
INDEX TO ADVERTISEMENTS ........ xii
ITEMS OF INTEREST ........ xi

